# Physical Activity, Sedentary Behavior and Sleep Time: Association with Cardiovascular Hemodynamic Parameters, Blood Pressure and Structural and Functional Arterial Properties in Childhood

**DOI:** 10.3390/jcdd8060062

**Published:** 2021-05-31

**Authors:** Mariana Gómez-García, Daniel Bia, Yanina Zócalo

**Affiliations:** 1 Departamento de Educación Física y Salud, Instituto Superior de Educación Física, Universidad de la República, Parque José Batlle y Ordoñez S/N, Montevideo 11600, Uruguay; mgomez@isef.edu.uy; 2 Departamento de Fisiología, Facultad de Medicina, Centro Universitario de Investigación, Innovación y Diagnóstico Arterial (CUiiDARTE), Universidad de la República, General Flores 2125, Montevideo 11800, Uruguay

**Keywords:** aortic pressure, arteries, blood pressure, cardiovascular risk factors, children, movement behaviors, physical activity, sedentary behavior, sleep

## Abstract

An association between movement behavior (MB) components (sleep time (ST), physical activity (PA) and sedentary behavior (SB)) and the state of the cardiovascular (CV) system in children has been postulated. However, it is still controversial whether MB components and/or sub-components (domains) during childhood are independently associated with aortic and peripheral blood pressure (BP), and structural or functional arterial properties. Aims: (1) to evaluate MB components and subcomponents associations with CV characteristics, (2) to analyze the explanatory capacity of interindividual variations in MB on CV properties inter-individual variations at the beginning of school age. Methods: Anthropometric, aortic and peripheral BP, hemodynamic levels (cardiac output, systemic vascular resistances), wave reflection indexes, and arterial structural (diameter, intima–media thickness) and functional (blood flow velocities, Doppler-indexes, local and regional arterial stiffness) parameters of elastic (carotids), transitional (brachial) and muscular (femoral) arteries and time spent in MB (PA questionnaires) were assessed in 816 children (5–6 years). Cardiovascular variables were standardized (z-scores), using age- and sex-related mean values and standard deviations obtained from subjects non-exposed to CV risk factors (CRFs) and who complied with 24 h MB recommendations (reference subgroup). Multiple linear regression models were constructed considering the CV z-scores as dependent variables and CRFs and MB components and subcomponents as independent variables. Results: CV variables showed independent association with MB variations. However, their explanatory capacity on CV characteristics was lesser than that of anthropometric indexes, sex and/or high BP. Conclusions: MB components and sub-components were associated with CV characteristics regardless of other factors, but their capacity to explain variations was lesser than that of anthropometric data, sex or high BP state. MB subcomponents (e.g., sedentary play and screen time in case of SB) showed different (even opposite) associations with CV parameters. ST was associated mainly with indexes of the ventricle ejective function, rather than with CV structural characteristics. SB component and subcomponents were associated with BP, but not with structural parameters. PA component and subcomponents were associated with both BP and structural parameters. The different arterial types, as well central and peripheral parameters showed independent associations with MB components and subcomponents. None of these were independently associated with arterial stiffness.

## 1. Introduction

In adults, an active lifestyle has been shown to be associated with a reduction in cardiovascular (CV) morbidity and mortality. Beneficial effects were initially thought to be associated to changes (improvement) in traditional cardiovascular risk factors (CRFs) profile. However, this could explain only about half of the CV risk reduction associated with an active lifestyle [1]. Then, movement behavior (MB) would also have direct effects on adults CV system [2]. MB can be characterized on the basis of data on its components, namely physical activity (PA), sedentary behavior (SB) and sleep time (ST). In this respect, recently, the 24 h movement guidelines established measurable recommendation thresholds for MB components considering children, adolescents and adults separately [3,4,5]. Compliance with guidelines recommendations was associated with better ‘critical health indicators’ (e.g., adiposity, cardiometabolic biomarkers, physical fitness, emotional regulation, psychological distress, behavioral conduct, cognition, quality of life/well-being, bone density, motor skills and self-esteem) [6,7,8,9]. However, the aim is to determine whether levels of PA, SB, ST and/or their sub-components (e.g., screen time, sedentary play for SB, active transport (walking, cycling), physical education at kindergarten for PA) are independently associated with CV status in childhood itself.

The associations between CRFs (e.g., obesity, blood pressure (BP)) and CV properties in children have shown dependence on the CV parameter (e.g., structural vs. functional) and vascular territory (e.g., elastic vs. muscular arteries, central vs. peripheral vessels) considered [10,11,12,13,14]. Based on this, it could be postulated that something similar might occur with the association between the MB and the CV system in children. Regarding this, data regarding the relationship between MB and CV status are not coincident but show divergences associated with (i) the arterial type or recording site (e.g., carotid vs. abdominal aorta [15], large vs. small arteries [16]), (ii) the MB sub-component analyzed [17,18] and/or (iii) the exposure to CRFs [15]. However, to our knowledge, to date there are no works that have comprehensively analyzed the association between MB and CV status in children, considering (i) hemodynamic variables (e.g., cardiac output, systemic vascular resistances), (ii) peripheral and central measurement (e.g., BP levels), (iii) wave reflections’ contribution to recorded BP and (iv) structural (intima–media thickness and diameters) and functional (e.g., local and regional arterial stiffness) arterial properties, analyzing different vascular territories.

This work’s aims were (i) to evaluate the strength of association of MB components (PA, SB, ST) and subcomponents, with CV properties, including cardiac output, systemic vascular resistances, central and peripheral BP levels, aortic wave analysis-derived components and structural and functional properties of elastic, transitional and muscular arteries and (ii) to analyze the capacity of interindividual variations in MB to explain interindividual differences in CV parameters in childhood (at the beginning of school age).

## 2. Materials and Methods

### 2.1. Study Population

The study was carried out in the context of CUiiDARTE Project [14,19,20,21]. The protocol was approved by the Institutional Ethics Committee (Comité de Ética en Investigación, Centro Hospitalario Pereira Rossell; Ethical approval: 29112013/29122015). Both parents’ written consent and children’s assent were obtained prior to the evaluation. The cohort (*n* = 816) was defined based on probabilistic, bi-stage and stratified sampling of subjects attending public kindergartens in Montevideo. It is a sub-sample of the population included in the longitudinal study ‘Patrón de crecimiento, estado nutricional y calidad de alimentación en la primera infancia: análisis de su impacto sobre la estructura y función vascular y el riesgo cardiovascular relativo en niños uruguayos’ (CUiiDARTE-Agencia Nacional de Investigación e Innovación (ANII), Ministerio de Desarrollo Social (MIDES), United Nations Children’s Fund (UNICEF)), which started in 2010 and had a second phase in 2016.

The study approach consisted in clinical and anthropometric assessment, questionnaires on MB, lifestyle and family history and a non-invasive CV evaluation.

### 2.2. Clinical and Anthropometric Evaluation

Anthropometric data (body weight (BW) and height (BH)) at birth were obtained from health control records (mandatory within the first 0–36 months (m) of life according to Health Ministry regulations) and/or from self-reports documented during interviews with parents [10]. Current BW (electronic scale, 841/843, Seca Inc., Hamburg, Germany; model HBF-514C and Omron Inc., Chicago, IL, USA) and BH (portable stadiometer) were measured with the participants wearing light clothing and no shoes. Considering measurements from our technicians and data from the health controls, we obtained the BW and BH values corresponding to 0 m (birth) and 6 years (y). BW for BH and body mass index (BMI; BW-to-squared BH ratio) were calculated. Then, using World Health Organization software (Anthro-v.3.2.2; Anthro-Plus-v.1.0.4), z-scores for males and females were obtained for different anthropometric variables (i.e., z-BW, z-BH, z-BMI, z-BW for BH). z-BMI was also calculated at the time of the CV study [10].

None of the included subjects were taking medications, had congenital, chronic or infectious diseases at the time of the CV evaluation. Clinical and anthropometric evaluations enabled to assess exposure to CRFs. Hypertension, dyslipidemia and diabetes were considered present if they had been previously diagnosed [10]. Subjects who had brachial artery systolic and/or diastolic BP (baSBP, baDBP) > 95th percentile for sex, age and BH during the study were considered with high BP levels. Obesity was defined as z-BMI ≥ 2. A family history of CV disease was defined by presence of first and/or second-degree relatives with early (<55 y: males; <65 y: females) CV disease.

### 2.3. Cardiovascular Evaluation

Cardiovascular studies were performed at the educational centers. Evaluations were carried out after at least 10 min of rest in supine position in a quiet, temperature-controlled room, which enabled reaching steady BP and heart rate conditions.

### 2.4. Peripheral and Central Pressure and Aortic Wave-Derived Parameters

Heart rate, baSBP, baDBP and tibial artery systolic (taSBP) and diastolic BP (taDBP) were obtained at 5 min intervals (Hem-4030, Omron Inc., Hoffman Estates, IL, USA). Brachial and tibial pulse pressure (PP) were calculated: baPP = baSBP − baDBP; taPP = taSBP − taDBP. Mean BP (MBP) was calculated as: MBP = baDBP + baPP/3. The Ankle Brachial Index (ABI; marker of central–peripheral BP amplification) was calculated as: ABI = taSBP/baSBP [22].

Subendocardial viability ratio (SEVR), systolic, diastolic and pulse central aortic BP (aoSBP, aoDBP and aoPP); wave reflection indexes (e.g., augmentation index (AIx) and forward (Pf) and backward (Pb) BP components, were assessed (random order) using two commercially available devices: SphygmoCor-CvMS ((SCOR); v.9, AtCor Medical, Australia) and Mobil-O-Graph PWA monitor system ((MOG); I.E.M.-GmbH, Stolberg, Germany) [23]. Radial and carotid BP waves were obtained by applanation tonometry (SCOR device). Acquired waves were calibrated to MBP and baDBP (HEM-433INT; Omron Healthcare Inc., Lake Forest, IL, USA). Aortic BP (aoBP) waves were derived from radial recordings using a general transfer function. Radial artery systolic, diastolic and pulse pressure (raSBP, raDBP, raPP, raPP = raSBP − raDBP) were obtained. Carotid artery pulse waves (carotid applanation tonometry) were supposed to be equal to aortic BP waves (due to the proximity of the arterial sites). Thus, a general transfer function was not applied to obtain central waves from carotid records. The aoSBP, aoDBP and aoPP levels were quantified from the obtained aoBP waves. Considering a triangular flow model, Pf and Pb components were separated [23]. Only accurate waveforms on visual inspection and high-quality recordings (in-device quality index > 75%) were considered. Additionally, baBP levels and waves were obtained with MOG (brachial cuff-based oscillometric device) [23]. The system determined aoBP levels and waves from brachial recordings (general transfer function). Then, by means of pulse wave and wave separation analysis, Pf and Pb were obtained [23]. Only high-quality records (index ≤ 2) and satisfactory waveforms (visual inspection) were considered.

First (P1) and second (P2) peaks in the aoBP wave were identified. Then, their height (amplitude) and time were determined. The difference between P2 and P1 was computed as central augmented pressure (AP), used to quantify aortic AIx (AIx = AP/aoPP). AIx depends on heart rate. Thus, AIx adjusted to 75 beats/minute (AIx@75) was calculated [23]. It is worth noting that AIx is a measure of reflections contribution to aoBP. It depends mainly on timing and magnitude of the reflected (backward) wave and is influenced by the stiffness and structure of vessels distal to the recording site, as well as by the distance to reflection sites and left ventricle function. Basically, greater Pb and/or AIx values indicate increased net and relative reflections, respectively, and/or earlier return of reflected waves due to increased stiffness and/or closer reflection sites.

The use of different recordings and approaches to assess aoSBP, Pf, Pb, SEVR and AIx@75 (i.e., brachial artery oscillometry vs. applanation tonometry; carotid vs. radial tonometry), is justified or explained by the fact that although ‘the same parameters’ are obtained, the physical-mathematical models underlying their calculations are not the same and have shown (in some cases) differences in the values they allow to arrive at [23]. Thus, aiming at minimizing the risk of bias in our findings, we opted for using more than one device and approach to assess some CV parameters (e.g., those for which yet there is not an approach considered the ‘gold standard’). Data from the different devices/approaches are shown and analyzed comparatively.

Systemic vascular resistances, cardiac output and cardiac index were quantified from brachial pulse contour analysis (MOG, I.E.M.-GmbH, Stolberg, Germany) [24]. Subjects’ values are the average of at least six records obtained in a single visit.

### 2.5. Regional Arterial Stiffness: PWV (cfPWV and crPWV) and PWV Ratio

Carotid-femoral (aortic) and carotid-radial (upper arm) pulse wave velocity (cfPWV and crPWV), markers of regional arterial stiffness, were assessed (applanation tonometry, SCOR, AtCor-Medical, Sidney Australia). cfPWV and crPWV values depend on the algorithm used to detect the so-called ‘foot of the wave’ and on the pathway considered [21,25]. This can be the ‘direct distance’ between recording sites (e.g., carotid and femoral) or the distance obtained by subtracting the carotid measurement site to the sternal notch distance from the sternal notch to peripheral measurement site distance (e.g., femoral) [25]. Following international recommendations, we used the direct distance multiplied by 0.8 for cfPWV (which enabled obtaining the real cfPWV). In turn, we considered the subtracted distance for crPWV [25]. cfPWV and crPWV values were obtained as the median of three measurements (random order).

Arterial stiffness is influenced by BP levels during the examination, which, if not considered, could lead to inaccurate conclusions. To overcome this problem, we calculated β-PWV= (Ln(baSBP/baDBP)) × ((PWV^2^ × 2ρ)/baSBP − baDBP)), where Ln is natural logarithm, PWV is cfPWV or crPWV and ρ is blood density (assumed 1060 kg/m^3^) [21]. Brachial BP, PWV and ρ were entered into the equation in Pa, m/s and kg/m^3^, respectively. β-PWV was suggested to better reflect structural changes of the arterial wall (with independence of arterial distending BP). Finally, PWV Ratio and β-PWV Ratio, an index of central-to-peripheral arterial stiffness gradient, was quantified as cfPWV/crPWV and βcfPWV/βcrPWV [21,25,26].

### 2.6. Arterial Diameter, Intima-Media Thickness and Local Arterial Stiffness

Left and right common carotid artery (CCA) and femoral artery (CFA) and left brachial artery (BA) were analyzed using ultrasound (6–13 MHz, M-Turbo, Sonosite Inc., Bothell, WA, USA). Sequences of images (30 s, B-Mode, longitudinal views) were stored for off-line analysis. Beat-to-beat diameter waves were obtained using border detection software. Peak systolic and end-diastolic diameters (SystD, DD) as well as intima–media thickness (IMT; far wall, end diastole) values were obtained averaging at least 20 beats [22]. CCA diameter and IMT were measured a centimeter proximal to the bulb; CFA diameter and IMT were measured in a straight segment in the penultimate centimeter proximal to the bifurcation and BA measurements were obtained at elbow level in a straight segment of at least one centimeter [22].

Local stiffness was quantified by pressure-strain elastic modulus (EM) and Stiffness Index (β; a ‘BP-independent’ index) [25]: EM = (SBP − DBP)/((SystD − DD)/DD); β = Ln(SBP/DBP)/((SystD − DD)/DD). aoSBP and aoDBP values were used to quantify CCA stiffness, whereas baSBP and baDBP were used to assess CFA and BA stiffness.

### 2.7. Carotid, Femoral and Brachial Doppler-Derived Blood Velocity

Peak systolic (PSV), mean, end-diastolic (EDV) and peak reversal (for CFA and BA) velocity levels were computed from CCA, CFA and BA blood velocity waves (Doppler Mode, 6–13 MHz, M-Turbo, Sonosite Inc, Bothell, WA, USA). Doppler-derived resistive, pulsate and systo-diastolic indexes were calculated.

### 2.8. Movement Behavior Evaluation

**Figure 1:**
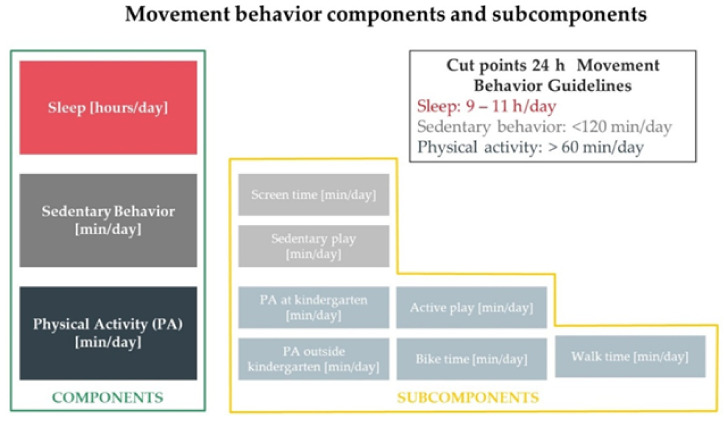
Movement behavior components and subcomponents (domains) measurement and cut-off points according to 24 h Movement Behavior Guidelines.

From a movement perspective, time in a 24 h period is divided (distributed) into PA, SB and ST. In our work, MB components were assessed by a pediatrician in an interview with the responsible adults (parents or legal tutors), using a questionnaire developed in agreement with national and international guidelines. The selected interviewer-administered questionnaire was found to be a valid and reliable assessment tool in children, as evidenced by its overall good correlation with accelerometry measurements, and was used in previous cross-sectional and longitudinal works [27,28,29]. The questionnaire was used to gather information pertaining to the child’s participation in physical activity across several domains (subcomponents) (Figure 1):

SB (outside of the kindergarten) was quantified as the sum (min/day) of (i) sedentary play (e.g., time spent reading, drawing, doing homework, playing a musical instrument or board games) and (ii) screen time (e.g., time spent watching television or videos, playing video/computer games and internet surfing). ST was quantified as the sum of night-time sleep and naps. Total PA was calculated as the sum of several domains: (i) PA at kindergarten, as part of physical education classes (defined as any exercise class supervised by a teacher during kindergarten time), (ii) PA outside of kindergarten defined as any extramural organized sport activity (e.g., in sports clubs, soccer teams), (iii) time of active play, which refers to PA outside of kindergarten, not as part of an organized sport activity (e.g., skipping, running, traditional games, playing with a ball) and (iv, v) biking and walking times (i.e., walking or biking to and from the kindergarten). The amount of each component and subcomponent was calculated by multiplying or adding the reported frequencies and times of the different activities. Based on the described parameters, we calculated children’s weekly (min/week) and daily (min/day) PA (Table 1).

Compliance with 24 h movement international recommendations was analyzed [3]. A child was deemed to comply with the recommendations if calculated times were (i) <120 min/day for SB, (ii) 9–11 h/day for ST and (iii) ≥60 min/day for moderate-to-vigorous PA [3]. PA intensity was classified based on values from compendia of energy costs, specifically developed for youth [30,31,32]. To estimate the intensity of each PA, <2 and ≥4 metabolic equivalent (MET) values were considered upper bound and lower bound for sedentary and moderate-to-vigorous intensity PA, respectively [33].

To improve the characterization of children’s MB, they were classified according to the time spent on (i) sedentary activities (>240, 180–240, 120–180 or <120 min/day), (ii) sleeping (<9, 11–13, 9–11 or >13 h) and (iii) moderate-to-vigorous PA (<30, 30–60, 60–90 or >90 min/day) (Table 1).

### 2.9. Data Analysis

A stepwise analysis was performed. First, descriptive statistics were obtained (Table 1 and Table 2). Second, CV variables were expressed as z-scores. To this end, subjects non-exposed to CRFs and who complied with 24 h movement recommendations were selected (reference subgroup) (Table S1). Working with the reference subgroup, age- and sex-related mean values (MV) and standard deviations (SD) were determined for each variable. Individual data were converted into z-scores, obtained by subtracting the reference MV to the measured value, dividing the result by the reference SD. Tables S2 and S3 show z-scores for the reference subgroup and for all the studied children. z-scores for the different CV variables showed a wide range of variation.

Third, multiple linear regression models (input: stepwise) were constructed considering the CV z-scores as dependent variables and MB and CRFs as independent variables (Table 3, Table S4). A variance inflation factor <5 was selected to evaluate (discard) significant multicollinearity.

Aimed at providing data about non-adjusted associations, bivariate correlations were analyzed. The results are included in Supplementary Materials Tables S5–S9. Specifically, we analyzed the association between (i) MBs levels and compliance with recommendations (Table S5), (ii) MBs levels and exposure to CRFs (Table S6), (iii) CV parameters and CRFs (Table S7), (iv) MB levels and CV z-scores (Table S8) and (v) CV z-scores and CRFs (Table S9). Two-tailed simple bivariate correlations (continuous variables) and point bi-serial correlations (continuous and dichotomous variables) were always performed. An association was considered significant only if the 95% confidence interval of Pearson’s coefficient, quantified by Bootstrapping, did not contain the 0 value. Bootstrap-derived 95% confidence intervals (1000 samples) were obtained applying bias-corrected and accelerated methods for computing confidence interval limits.

Fourth, by using (i) multiple linear regression models non-standardized β coefficients, (ii) MV and SD data (reference subgroup) and (iii) minimum and maximum values for each MB variable (variation range), it was possible to quantify for the different CV variables (in their respective units) (1) the maximum variation (in our group of children) that could be associated (attributed) to different values obtained for MB components or subcomponents and (2) the variations that could be (theoretically) expected considering inter-individual variations in time spent on SB, ST and PA (and their subcomponents). Data are shown in Table 4 (ordered by the CV parameter) and Table S10 (ordered by MB component and subcomponent).

According to the central limit theorem, a normal distribution was considered (taking into account Kurtosis and Skewness coefficients distribution and number of studied subjects with sample size >30) [34]. The number of subjects studied (*n* = 816) was higher than the minimum number calculated for α = 0.05, β = 0.20 and a hypothesized or anticipated correlation coefficient (r) equal to 0.1 (*n* = 782), 0.5 (*n* = 29) or 0.9 (*n* = 7). Analyses were carried out using SPSS Software (v.26, IBM-SPSS Inc., Chicago, IL, USA). A *p* < 0.05 was considered statistically significant.

## 3. Results

### 3.1. Movement Behavior, Blood Pressure and Arterial Characteristics’ Associations

We found that in children 5–6 y old, variations in MB components and/or subcomponents contributed to explain cardiac output, central BP, peripheral BP and arterial structural and functional parameters’ deviations from expected (reference mean) values, regardless of (i) demographic characteristics (age, sex), (ii) current and at birth anthropometric data (z-BW, z-BH, z-BW for BH, z-BMI) or (iii) exposure to traditional CRFs (e.g., high baBP, obesity). At the same time, the explanatory capacity was independent of the other MB components and subcomponents (Table 3 and Table 4). Some associations between MB levels and CV properties depended on exposure to traditional CRFs, since they ‘disappeared’ when passing from simple bivariate to multiple linear models. As a general rule, CV variations were explained by anthropometric data (e.g., z-BMI), followed by sex and high BP and finally by MB characteristics (relative contribution order from highest to lowest, according to standardized β) (Table 3).

All arterial territories (elastic, transitional and muscular arteries; central and peripheral territories) were sensitive to variations in MB components or sub-components (Table 3 and Table 4). However, results showed that (i) ST were mainly associated with ‘central’ variables, particularly with the ventricle ejective function (cardiac output and Pf), rather than with arterial (structural or functional) properties, and (ii) SB component and subcomponents were associated with BP (aoBP, baBP and taBP) levels, but not with structural parameters. In turn, (iii) PA component and subcomponents were associated with BP as well as with the structural properties of the different arterial types. On the contrary, none of MB components and sub-components showed association with local or regional arterial stiffness (disregard of the arterial territory analyzed) (Table 3, Table S10).

### 3.2. Sedentary Behavior and Cardiovascular Properties: Independent Associations

Longer screen times were positively associated with z-baDBP, z-aoDBP and z-AIx@75(MOG) levels. Regardless of other factors, variations in screen time (considering data observed in our population) explain variations of 7.27% in AIx@75(MOG), 2.7 mmHg in baDBP and 3.6 mmHg aoDBP, which represent variations of 33.3%, 4.6% and 6.1%, respectively (considering reference values) (Table 3 and Table 4, Table S10).

Sedentary play was not associated with z-baBP or z-aoBP, but it was negatively associated with z-taBP and z-AIx@75 (CT). Sedentary play explains variations up to 4.05 mmHg in taDBP, 6.25 mmHg in taSBP and 11.86% in AIx@75(CT), which represent variations of 6.97%, 5.50% and 229%, respectively (Table 3 and Table 4, Table S10).

SB (min/day) was not associated with z-BP but was negatively associated with z-ABI. A maximum variation in ABI equal to 5.43% could be explained by differences in SB times observed in our group of children (Table 3 and Table 4, Table S10).

### 3.3. Sleep Time and Cardiovascular Properties: Independent Associations

ST was negatively associated with Pf (CT) and cardiac output; differences in ST explain variations equal to 12 mmHg and 0.37 L/min (42.5% and 8.4%), respectively (Table 3 and Table 4, Table S10).

### 3.4. Physical Activity and Cardiovascular Properties: Independent Associations

PA in kindergarten was negatively associated with taSBP, taPP, bSBP and aoSBP (RT) levels. In turn, active play, biking and total PA were positively associated with taPP, taDBP and taSBP, respectively.

Structural arterial characteristics showed positive associations with PA subcomponents: (i) PA at kindergarten was associated with CCA IMT, (ii) PA outside of kindergarten was associated with CCA diameters, (iii) total PA (min/week) was associated with CFA diameters and (iv) walking time (min/week) was associated with BA diameters. Differences in PA could explain variations equal to 0.011 mm in CCA IMT, 0.30 mm in CCA DD, 0.32 mm in CCA SysD, 0.54 mm in CFA SysD, 0.49 mm in CFA DD, 0.31 mm in BA DD and 0.32 mm in BA SystD, representing variations of 2.52%, 5.66%, 5.25%, 10.81%, 10.49%, 12.59% and 12.09%, respectively (Table 3 and Table 4, Table S10).

Blood velocities z-scores showed low levels of association with MB. Longer PA time at and outside of kindergarten were negatively associated with CCA and BA EDV levels. Walking time was positively associated with CFA PSV levels (Table 3 and Table 4, Table S10).

There were no significant associations between PA and arterial stiffness indexes. This was the case (i) before and after adjusting for BP, (ii) for both, local and regional arterial stiffness and (iii) when analyzing center-periphery stiffness gradient (Table 3).

## 4. Discussion

To our knowledge, this is the first study to evaluate the independent associations of PA, SB and ST and their subcomponents (domains) with the CV status in early life (5–6 y), analyzing in a large sample several arterial pathways and complimentary parameters using different non-invasive approaches (always considering the gold standard if available). This study showed that in children aged 5–6 y, ST, SB and PA are already associated with the CV system status (maybe exerting their initial direct effects). This would be the case for a given MB component, irrespective of the others and of the exposure to traditional CRFs.

### 4.1. Sedentary Behavior and Cardiovascular Inter-Individual Variations: Independent Associations

#### 4.1.1. SB and Central and Peripheral Blood Pressure

Our results showed that screen time was positively associated with baDBP (0.3 mmHg h/day), whereas time spent on sedentary play showed no association with baBP, but it was negatively associated with taSBP (0.8 mmHg h/day) and taDBP (0.5 mmHg h/day). Then, SB subcomponents would be associated with different BP characteristics and the associations would be the opposite.

At least three aspects should be highlighted when interpreting these results. First, works that examined the association between SB (mainly screen time) and BP in children arrived at dissimilar findings. Whereas some studies reported ‘positive associations’ (higher screen time, higher baBP) [35,36,37,38], others found that the associations were not significant [39,40]. This controversy could be explained, at least partially, by the existence of differences not only in the time spent on the activity (‘screen time’), but also in the time of day at which the exposition occurs [41]. Regardingthis, recently Pedersen et al. (2020), in a study of pre-school children followed over 2 y (*n* = 963), found no significant prospective association between ‘total screen time’ and baBP or prevalence of high BP. However, days per week with ‘screen time before bedtime’ (2–5 or ≥6 days/week) was independently and positively associated with the prevalence of high BP at age 5 y (e.g., covariates included BMI, sleep time, PA level) [41]. On the other hand, the dissimilar findings could also be explained by differences in kind of screen-related activity. In adolescents (aged 13–17 y), Martinez-Gomez et al. (2012) found that console videogames, but not computer games, were positively associated with a clustered cardiometabolic risk score, which included baDBP and MBP [42]. Unfortunately, in this work (like in many others), activities includingthe screen-related ones were not registered and classified according to their characteristics and time of the day in which they were carried out.

Second, in agreement with this work’s results, in previous works it was proposed that SB sub-components could show opposite associations with baBP. In a prospective study (5 y follow-up; *n* = 821, age: 6.7 y at baseline) Gopinath et al. (2014) reported that after adjusting for cofactors (e.g., age, baseline BH, BP, BMI, time in different PAs), (i) every h/day spent on screen activities was positively associated with baDBP (0.69 mmHg) and MBP (0.59 mmHg), while (ii) time spent ‘doing homework’ was negatively associated with baSBP (−1.12 mmHg, p at the significance umbral, 0.10) [43]. This agrees with the authors’ findings in adolescents (*n* = 2353; mean age: 12.7). In this group, after adjusting for cofactors, positive association between baDBP and every h/day spent on (i) screen activities (0.44 mmHg), (ii) watching TV (0.99 mmHg) or (iii) playing video games (0.64 mmHg) were reported. Similarly, significant positive associations were observed between screen activities, TV watching and MBP. However, (i) time spent reading was negatively associated with baSBP (0.91 mmHg for every h/day), baDBP (0.69 mmHg for every h/day) and MBP (−0.76 mmHg for every h/day) and (ii) time spent doing homework was negatively associated with baSBP (0.80 mmHg for every h/day) and MBP (0.51 mmHg for each h/day, *p* = 0.07). Additionally, every hour spent on homework was associated with a 19% reduction in the odds of having high BP [44]. The above agrees with our findings, since in this work, ‘time spent reading or doing homework’ were considered subcomponents of ‘sedentary play’, which was negatively associated with BP. Similarly, works that analyzed factors other than CV characteristics found that different SB subcomponents (e.g., reading, sitting, playing video games) and different SB patterns could differently impact health status. For example, screen time may be detrimental and reading beneficial to cognitive development in early years of life [45]. In addition, screen time, but not other SB activities, showed positive associations with z-BMI and waist circumference [46]. Then, data from Gopinath et al. (2012, 2014) [43,44] and this work suggest that whereas ‘screen time’ would be associated with increased baBP and aoBP, ‘sedentary play’ would not only not be associated with higher BP levels (brachial or aortic), but might even show association with lower BP values (in the tibial artery).

Finally, differences in SB patterns and levels between groups (sample characteristics) could contribute to explain the dissimilar findings. In the work of Chinapaw et al. (2014) (in which there was no association between screen time and BP), children had an average screen-time of 1.2 h/day, whereas in our work, children were exposed to screen-based activities for an average of 2.5 h/day. Considering the low screen times reported by Chinapaw et al., it may not have included children exposed to screen times above thresholds necessary to observe (or establish) an association with baBP [40].

Additionally, our results showed that higher screen times were positively associated with aoBP levels and AIx@75(MOG). Thus, the positive association between BP and time spent on screen activities would not only represent a peripheral phenomenon (e.g., increase in pulse amplification), but would also involve central arteries, determining a real increase in ventricle load. Taking into account the association between AIx@75 and screen time, it could be stated that an increase (relative) in reflected waves contribution to aortic pulse wave could be an explanatory factor for the increased aoBP observed in association with higher screen times. In this regard, it should be pointed out that pulse wave in a given arterial site results from integration of both incident and reflected components [25]. The above was not a homogeneous finding among the different approaches (e.g., it was not observed when using SCOR device). Further works are necessary to enhance our knowledge and comprehension of the physiological phenomenon that accounts for or underlies the association between SB aortic wave analysis-derived (levels and components) characteristics.

#### 4.1.2. SB and Arterial Structural and Functional Parameters

In this work, SB components or subcomponents showed no association with arterial structural and functional parameters.This is in agreement with previous works in which no association was reported between self-reported or objectively measured SB time and arterial stiffness or CCA IMT in children [47,48]. In primaryschool children (aged 6–8; *n* = 136), Haapala et al. (2017) reported no independent association between SB (accelerometry) and an arterial stiffness index (measured at the fingertip by pulse contour analysis) [47]. Similar findings were reported by the authors when applying questionnaires (instead of accelerometry) to the same cohort, which enabled to divide SB into (i) screen-based SB (e.g., watching TV, using a computer and playing video games, using a mobile phone) and (ii) other SB (e.g., listening to music, playing a musical instrument, reading, writing, drawing, arts and crafts, playing board games, resting) [48]. In agreement with the described above are findings reported by Nettlefold et al. (2012), who analyzed the association between SB (accelerometry) and large arteries compliance (*n* = 102, age: 8–11 y) [16]. In the same line are the results of Kochli et al. (2019), who used questionnaires to assess the association between screen time and arterial stiffness (oscillometry) in children (*n* = 1171, age: 6–8 y) [49]. The described findings showed that in children (aged 5–10 y), SB would be associated with CRFs and BP (as discussed above), but not with variations in structural and functional arterial properties (Table 3). The observations are consistent with the fact that structural and/or functional changes represent a slow process that requires long-term exposure to a given condition (e.g., unhealthy sedentary lifestyle).

### 4.2. Sleep Time and Cardiovascular Inter-Individual Variations

ST was negatively associated with cardiac output and forward (Pf) aoBP component. Thus, lower ST, higher cardiac output and incident aoBP component are determinants of BP levels. The referred factors could contribute to explain the negative association between ST and baBP reported in some works that did not include them in multiple regression analysis [50,51,52]. Regardingthis, in this work there was positive association between cardiac output and high baBP (Table 3). Then, at least in theory, the higher baBP levels associated with shorter ST could be explained by relative ‘hyperdynamic’ state, rather than by higher SVR and/or arterial stiffness levels. Similarly, in obese children, higher baBP levels were observed together with increased cardiac output and forward component of the pressure wave, but not with higher arterial stiffness or SVR [14].

In agreement with the hypothesis that ST would impact cardiac parameters (rather than arterial properties), Feng et al. (2016) reported an (independent) association between short ST and cardiac remodeling in adolescents (e.g., increased interventricular septum thickness, left ventricle end-diastolic diameter, posterior wall thickness, left ventricle mass and mass index). In addition, authors found a trend towards higher resting heart rates in subjects reporting short ST (≤7 h/night). Consequently, in adolescents, a reduced (insufficient) ST could be a factor independently associated with deleterious structural cardiac changes, which in turn could be related to an hyperdynamic state (as observed in obese children) [53].

In this work, ST was not associated with structural and functional arterial parameters.Thus, for at least at 5–6 years of life, ST would be associated with cardiac parameters rather than with vascular variations.

### 4.3. Physical Activity and Cardiovascular Inter-Individual Variations

#### 4.3.1. PA and Central Pressure, Peripheral Pressure and Blood Flow Velocities

In this work, PA at kindergarten was negatively associated with taSBP (2 mmHg per each h/week), taPP (1.5 mmHg per each h/week), baSBP (1.1 mmHg per each h/week) and aoSBP (RT) (1.7 mmHg per each h/week). In general terms, the findings agree with previous works that showed a negative association between PA and baBP [54,55,56]. However, it isworth noting that similar to that reported by Gopinath et al. (2011), in this work, the association was only observed for PA at kindergarten. In their work (*n* = 1765, age: 6.7 ± 0.4 y), Gopnath et al. found that after adjusting for cofactors (e.g., age, sex, BH and BMI), time spent on outdoor activities was not associated with baBP, whereas indoor activities were negatively associated with baDBP (2.4 mmHg per each h/week) and MBP (2.2 mmHg per each h/week) [56]. The finding that PA at kindergarten was the single PA subcomponent associated with (the expected) negative trend towards lower BP levels could be explained by the fact that subcomponent could be the most regular, structured (and objectively measurable) activity developed, ableto classify children based on time spent on the specific PA (0, 30, 60 or 90 min/week). PA at kindergarten would be mainly an ‘indoor’ activity, which increases the probability of reaching high levels of intensity. About this, it was stated that compared to outdoor activities (e.g., soccer), those developed indoors (e.g., soccer, basketball) would involve shorter sessions of more intense activity since indoor sports are typically played on smaller areas so that participants cover less total distance [56].

We found that active play, biking and total PA times were positively associated with taPP (0.2 mmHg per each h/week), taDBP (0.8 mmHg per each h/week) and taSBP (0.2 mmHg per each h/week), respectively. Further works would contribute to the understanding of the meaning and explanatory factors for this (original) finding. However, it could be proposed that an increased blood flow in lower limbs could contribute to the higher peripheral (tibial artery) BP levels observed in active children. In other words, the activity itself could conduct to an adaptive CV response to accurately fulfill the metabolic necessities (e.g., associated with increased muscular mass and/or requirements). In this context, it is worth noting that PA subcomponents were associated with larger CFA diameters and PSV, which would result in larger CFA blood flow (blood flow = cross sectional area * blood flow velocity). On the other hand, PA subcomponents were also associated with higher CFA S-DIndex levels (indicators of local–regional resistances). Consequently, both ultrasound-derived (diameters, velocities, S-DIndex) and oscillometric-derived (taBA) data suggest that higher PA times would be associated with higher blood flow, blood pressure and vascular resistances levels in lower limbs. Once again, future works are necessary to contribute to define the described associations, as well as to deepen understanding oflocal hemodynamic phenomena that may occur in specific arterial territories during growth (e.g., as adaptive process).

#### 4.3.2. PA and Arterial Structural Parameters

PA (component or subcomponents) was associated with arterial structural characteristics. PA at kindergarten was positively associated with CCA IMT, whereas CCA, CFA and BA diameters were positively associated with PA out of kindergarten, total PA and walking time, respectively. These results are in agreement with the report by Ried-Larsen et al. (2013), who in a prospective study (*n* = 205, age: 9 y at baseline, 6y follow up) evaluated PA (accelerometry) and measured CCA IMT (at age 15 y (*n* = 254). Adjusted models showed positive (at significance umbral) relationships between CCA IMT and both mean PA intensity from childhood to adolescence and change in moderate-to-vigorous or vigorous PA intensity in the same period [17]. Additionally, in a previous work, the authors had found that children who rode bicycles every day showed a trend towards higher CCA IMT compared to those (boys) who rode <3 times/week [18]. The finding of a positive association between PA and CCA IMT might be considered unexpected, if we think of IMT in terms of its demonstrated association with CV risk and disease (e.g., atherosclerotic). However, as previously analyzed, PA during growth and development (e.g., in early lifestages such as childhood) might be associated to adaptive CV changes that could include an enlargement of the medial layer of the arterial wall. During high-intensity PA, shear-stress and cyclic strain are (acutely) increased. In turn, long-term exercise has shown to be associated with an enlarged arterial diameter and could also associate wall hypertrophy (e.g., IMT increase). According to ‘Law of Laplace’, wall stress relates positively with arterial diameter (radius) and negatively with wall thickness. Therefore, conditions associated with increased diameters may be counteracted by compensatory mechanisms in order to maintain the ‘arterial tensional homeostasis’. The above contributes to comprehension of the observed associations between PA and arterial structure (e.g., diameters and/or IMT). Nonetheless, it is worth noting that data on PA and CCA IMT relationship are not universal, but have shown differences. Regardingthis, opposite ofthe above-described, there are works that showed no association between the described parameters and works in which the association was negative. The dissimilar findings could be explained by differences in the PA subcomponent considered, in the cofactors analyzed and/or in the age of the subjects [2,15,57].

#### 4.3.3. PA and Arterial Functional Parameters

In this work, PA levels showed no significant association with wave reflections (e.g., AIx@75) or arterial stiffness (local, regional, central-to-peripheral stiffness ratio) levels, both before (ME and PWV) and after (β and β-PWV) adjusting for BP. This agrees with data from other works in which local stiffness, regional stiffness and/or AIx@75 were assessed in children (e.g., aged 5 and 8 y; 6–8 y; 8–11 y) and/or adolescents (12–14 y; 17 y; 15 y) [2,15,16,17,49,58].

There are also works that reported a negative linear association between arterial stiffness and some PA intensity levels. Haapala et al. (2017) studied the association between PA and cardio-respiratory fitness and a ‘global arterial stiffness index’in children (aged 6–8; *n* = 136). Moderate, vigorous and cumulative time spent in PA were inversely associated with stiffness. However, in subjects with PA <3 METS, PA was not associated with arterial stiffness [47]. Therefore, it could be said that PA would be associated with arterial stiffness once an intensity threshold is surpassed. In the same line, it could be said that PA and arterial stiffness association could depend on the distribution of PA intensities in the studied subjects.

Similarly, in boys (*n* = 169), Ried-Larsen et al. found that those who rode bicycles every day (not near every day) had lower arterial stiffness (lower EM, higher distensibility and compliance) than boys who rode <3 times/week. However, authors did not observe any differences in arterial stiffness across categories of bicycling in girls [18]. Finally, Vijalainen et al. (2016) reported that unstructured PA time (but not total PA time or time spent on other PA subcomponents) was negatively associated with stiffness (but not with reflection) indexes. The independent association disappeared when cofactors related with cardio-respiratory fitness were considered [48]. Unfortunately, stiffness indexes capable of characterizing intrinsic arterial properties with independence on BP (e.g., β index) were not assessed in available works. In this regard, it is worth noting that arterial pressure and diameter show a non-linear passive relationship. Thus, BP variations associate passive and non-linear changes in arterial diameter with resultant variations in arterial stiffness [21,25]. An accurate analysis of the impact of PA on the arterial stiffness would require not only considering adjusting data on a population basis (e.g., using multiple linear regressions), but also on an individual basis (e.g., adjusting for the subject BP levels during the study). However, such approaches are seldomused, which makes it difficult to define whether PA and arterial stiffness association would depend on (or be explained by) PA impact on BP (not eliminated when using multiple linear regressions).

### 4.4. Movement Behavior and Cardiovascular Properties: Independence and Impact on Different Arteries

We found that variations in MB components and/or subcomponents contributed to the explained deviations (from expected reference mean) of several CV variables, regardless of (i) demographic, (ii) current and at birth anthropometric data and (iii) exposure to traditional CRFs. At the same time, the explanatory capacity of a given MB component or subcomponent was independent of others. As was analyzed, the findings agree with results of previous works.

In this work, we found that all the arterial territories (elastic, transitional and muscular arteries; central and peripheral territories) were sensitive to variations in MB components or sub-components. Therefore, MB would be associated with characteristics of the CV system as a whole, rather than with properties limited to a specific component. However, our results also showed that in children aged 5–6 y, the associations between MB and the CV system would vary depending on the component and subcomponent of MB and/or on the CV variable considered. ST would be associated with parameters related with the ventricular ejective function (cardiac output, Pf) but not with the arterial function, and SB (component and subcomponents) would be associated with BP, rather than with arterial structural properties. On the contrary, PA components and subcomponents would show association with BP and with the structural characteristics of the different arteries. None of the MB components or subcomponents were associated with arterial stiffness.

Looking at our findings, it could be said that in a hierarchical order (defined from greater to lesser explanatory capacity), variations in CV parameters could be explained by (i) current anthropometric data, (ii) sex or high BP and (iii) by MB characteristics. Similarly, in previous works from our group, z-BMI at the time of CV evaluation showed the greatest capacity to explain variations in central and peripheral BP and CV (structural and functional) properties at 6 and 18 y old subjects. It should be noted that z-BMI explanatory capacity was higher than that of CRFs such asfamily history of CV, hypertension, dyslipidemia or smoking [10]. This agrees with works previously described in that after adjusting multiple association analyses for several cofactors, anthropometric (nutritional) characteristics mostly remain in the models, suggesting they could be a final common pathway (mediator) by which MBs are associated with the CV system. In this regard, it is worth noting that even remaining in the models, the relative contribution of MB (standardized β) would be mostly lower than that of anthropometric parameters.

### 4.5. Strengths and Limitations

This work has strengths and limitations that should be considered. First, as CV disease manifests in later life, studies in children can only be performed with outcomes such as CRFs levels, BP and CV characteristics as an estimate of early life CV ‘damage’ or deviation from expected levels for sex and age. Our work included a comprehensive non-invasive evaluation of CV properties, obtained from a relatively large population sample of children. Second, different methodological approaches were considered to assess CV parameters (e.g., wave reflection parameters, aortic BP) in order to not bias our results to a particular approach. By using different devices and approaches, we were able to analyze whether associations for a given parameter would vary (or not) depending on the approach or device considered. This would be particularly useful when analyzing parameters yet under research and/or for which the gold standard (technique and/or approach) is to be defined. Third, defining a reference subgroup made it possible to determine interindividual variations (z-scores) of CV variables. Since the reference subgroup included Uruguayan children non-exposed to CRFs, who complied with MB recommendations, we avoided using bibliographical data from children who do not necessarily present characteristics similar to those of the Uruguayan children. In this regard, it is worth noting that our group has identified differences in CV characteristics among subjects from different populations [21,24]. Fourth, the number of subjects and the statistical approach were designed in order to increase the reliability of the confidence intervals and to analyze the association between MB and CV characteristics with independence of exposure to CRFs and MB components or subcomponents other than those being evaluated. However, although we adjusted for several covariates, we cannot discard the possibility of residual confounding factors (e.g., parental activity levels, genetic variation and socio-cultural characteristics) that could have influenced our results. Fifth, in this work, central and peripheral SBP and DBP were used to quantify central and peripheral arterial stiffness levels. This should be considered a strength. In previous works, CCA stiffness was quantified using baPP, which could lead to inaccuracies that would be greater at lower ages [15,17].

We are aware that our research may have limitations. First, it is a cross-sectional study. Since children were not followed over time, the temporal profiles of the CV characteristics, the exposure to different CRFs and the time spent on MB components and subcomponents are unknown. Second, parent-filled questionnaires were used as a tool to collect data on MB, despite the fact there are methods that allow PA to be evaluated more objectively and independently of the operator (e.g., accelerometry). Consequently, the completeness and accuracy of information may have been influenced by how parents and/or teachers perceived the questions. However, it should be noted that other techniques or approaches proposed to assess MB have limitations, particularly in children. As an example, there are controversies regarding (i) where the accelerometer should be placed (wrist vs. waist), (ii) which would be the best functions (equations) to quantify ST vs. rest, (iii) PA thresholds that should be used to classify PA as light, moderate or vigorous, (iv) the minimum recording time that should be considered representative of PA and (v) the algorithms (e.g., epoch length, sampling frequency) that should be used to assess PA. Additionally, the activity monitor does not capture some activities carried out especially in the evaluated ages (e.g., bicycling) [17]. Considering the described controversies, economic cost and mainly the age and number of children to be evaluated and the work design, we opted for assessing MB working with validated questionnaires elaborated based on international recommendations. Third, the age range was small. Thus, a robust analysis in terms of association between components and subcomponents of MB and children age was not possible. However, the small age range could also be considered as a strength for the analysis, avoiding effects on the results due toage differences. Finally, in this work, sex was considered when determining mean values and standard deviations (reference subgroup) used to typify (z-scores) CV data and when defining regression models (sex was introduced as an independent variable). The first is a very important issue since sex-related differences in CV parameters could be observed not only in mean values, but also in standard deviations. On the other hand, sex was included in the regression models as a co-factor. In this, we ensure that sex is not globally influencing the results obtained. However, it is worth noting that we did not perform ‘moderation/mediation’ analysis to evaluate whether associations between MB behavior components and CV z-scores varied depending on sex and/or other variables (e.g., z-BMI, ST, PA, SB). In this opportunity, we opted for not including such a sub-analysis.

## 5. Conclusions

Variations in MB components and/or subcomponents contributed to explaining the deviation of CV variables from expected values, regardless of demographic, anthropometric, other MB components/subcomponents data and exposure to CRFs. All the arterial territories (elastic, transitional and muscular arteries; central and peripheral) were sensitive to variations in MB components or sub-components. As a general rule, according to a hierarchical order (defined from greater to lesser explanatory capacity), variations in CV parameters could be explained by (i) current anthropometric data, (ii) sex or high BP and (iii) MB characteristics.

When considering SB, screen time was positively associated with baDBP, whereas time spent on sedentary play showed no association with baBP and was negatively associated with taBP. SB subcomponents showed different associations (and even in the opposite direction) with different BPs. Longer screen times were positively associated with aoBP and AIx@75(MOG). SB was not associated with arterial structural and functional parameters.

ST was negatively associated with cardiac output and forward (Pf) aoBP component.

PA at kindergarten was negatively associated with taSBP, taPP, baSBP and aoSBP (RT). In contrast, time of active play, biking time and total PA time were positively associated with taPP, taDBP and taSBP, respectively. PA at kindergarten was positively associated with CCA IMT, while CCA, CFA and BA diameters were positively associated with PA outside of the kindergarten, total PA and walking time, respectively. Thus, PA (components or sub-components) showed association with structural arterial characteristics. On the contrary, PA showed no association with reflection parameters or arterial stiffness.

## Figures and Tables

**Table 1:** Demographic, anthropometric, clinical and movement behavior (components and sub-components) data.

	MV	SD	Min	p25th	p50th	p75th	Max
**Demographic, anthropometric and factors related with increased cardiovascular risk**							
Age (years)	5.83	0.35	4.62	5.58	5.84	6.10	6.60
Body weight (Kg)	22.14	4.35	13.95	19.20	21.10	24.10	43.40
Body height (cm)	113.9	5.4	99.0	110.2	113.9	117.2	140.0
z-Body weight for height at birth (SD)	0.39	1.14	−4.06	−0.27	0.45	1.07	4.64
z-Body height for age at birth (SD)	−0.47	1.33	−6.12	−1.15	−0.35	0.46	4.75
z-Body weight for age at birth (SD)	−0.22	1.24	−5.98	−0.77	−0.07	0.61	2.56
z-Body mas index for age at birth (SD)	0.13	1.21	−7.00	−0.55	0.24	0.85	3.65
Current z-Body mass index (SD)	0.90	1.20	−3.03	0.09	0.70	1.59	5.00
baSBP percentile	68	19	3	57	72	84	99
baDBP percentile	64	16	17	53	64	75	99
High blood pressure state (%)	14.3						
Family history of cardiovascular disease (%)	36.9						
Diabetes (%)	0.0						
Dyslipidemia (%)	0.0						
Hypertension (%)	0.0						
Obesity (%)	16.6						
**Physical activity, sedentary behavior and sleep time**							
Sedentary play (min/day)	108	80	0	60	90	140	480
Screen time (min/day)	151	105	0	80	120	180	600
Sedentary behavior (min/day)	255	133	0	150	240	330	690
Sleep time (hours/day)	10	1	7	9	10	11	17
PA at kindergarten (min/week)	40	34	0	0	40	70	90
PA out of kindergarten (min/week)	30	66	0	0	0	0	260
PA: active play (min/week)	238	293	0	0	120	350	1200
PA: biking (min/week)	19	49	0	0	0	0	250
PA: walking (min/week)	104	109	0	30	75	150	500
Total PA (min/week)	448	365	0	190	350	600	1800
Total PA (min/day)	64	52	0	27	50	86	257
**International recommendations compliance**							
Sedentary behavior (%)	18.00						
Sleep time (%)	75.60						
Total weekly PA (%)	41.60						

MV: mean value. SD: standard deviation. Min., Max.: minimum and maximum. p25th, p50th, p75th: percentile 25, 50 and 75. z: z-score. PA: physical activity. SBP, DBP: systolic and diastolic blood pressure.

**Table 2:** Central and peripheral blood pressure, structural and functional arterial characteristics.

	MV	SD	Min	p25th	p50th	p75th	Max
**Central and peripheral blood pressure, heart rate and hemodynamic parameters**							
aoSBP (mmHg)	86	6	69	82	86	90	103
aoDBP (mmHg)	60	6	45	57	60	64	82
aoPP (mmHg)	26	5	14	22	26	29	51
baSBP (mmHg)	99	6	80	95	99	104	121
baDBP (mmHg)	59	5	45	56	59	62	81
baPP (mmHg)	40	5	24	36	40	44	60
taSBP (mmHg)	115	8	92	110	115	120	140
taDBP (mmHg)	60	6	44	56	60	64	81
taPP (mmHg)	55	7	29	50	55	60	76
raSBP (mmHg)	94	9	71	88	93	100	121
raDBP (mmHg)	59	6	45	55	59	62	81
raPP (mmHg)	35	8	19	29	34	40	69
Heart rate (beats/minute)	92	11	58	84	91	99	134
Ankle Brachial Index	1.16	0.08	0.96	1.1	1.15	1.2	1.45
Cardiac output (L/min)	4.24	0.47	3.1	3.9	4.22	4.5	6.17
Systemic vascular resistances (mmHg/L/min)	1.15	0.11	0.82	1.08	1.15	1.22	1.45
Cardiac Index (L/min/m^2^)	5.15	0.67	3.4	4.67	5.1	5.58	7.85
**Common carotid artery (CCA)**							
CCA SystD (mm)	5.96	0.45	4.84	5.64	5.97	6.24	7.45
CCA DD (mm)	5.29	0.42	4.28	4.97	5.29	5.57	6.95
CCA IMT (mm)	0.42	0.02	0.36	0.41	0.42	0.43	0.52
CCA EM (mmHg)	208	51	101	177	205	237	447
CCA β	2.88	0.71	1.31	2.39	2.8	3.31	5.87
CCA PSV (cm/s)	129	21	82	116	127	141	209
CCA EDV (cm/s)	34	6	17	30	34	38	57
CCA RI	0.74	0.04	0.59	0.71	0.74	0.76	0.86
CCA PI	1.73	0.27	1.09	1.54	1.7	1.88	2.71
CCA S-D Index	3.91	0.69	2.44	3.44	3.83	4.27	7.17
**Common femoral artery (CFA)**							
CFA SystD (mm)	4.72	0.49	3.6	4.39	4.72	5.01	6.37
CFA DD (mm)	4.40	0.49	3.26	4.08	4.38	4.68	5.92
CFA IMT (mm)	0.33	0.03	0.27	0.32	0.33	0.35	0.41
CFA EM (mmHg)	606	222	291	446	572	697	1978
CFA β	7.78	2.81	3.46	5.96	7.28	8.9	23.57
CFA PSV (cm/s)	125	25	71	108	123	142	231
CFA EDV (cm/s)	−17.88	15.8	−55.3	−26.4	−20.2	−13	41.85
CFA RI	0.98	0.11	0.65	0.91	0.94	1.05	1.8
CFA PI	5.49	3.49	1.71	3.35	4.52	6.35	29.15
CFA S-D Index	29.81	42.21	−0.34	9.03	11.2	15.63	192.3
**Brachial artery (BA)**							
BA SystD (mm)	2.5	0.31	1.49	2.31	2.48	2.69	3.52
BA DD (mm)	2.33	0.31	1.39	2.1	2.32	2.5	3.32
BA EM (mm)	572	214	210	420	521	687	1583
BA β	7.33	2.61	3.21	5.59	6.72	8.74	19.58
BA PSV (cm/s)	100.13	23.69	49	82.2	98.1	116.6	191.1
BA EDV (cm/s)	9.76	8.67	−20.9	7.21	10.75	14.4	33
BA RI	0.91	0.09	0.76	0.86	0.89	0.92	1.27
**Regional arterial stiffness**							
cfPWV (m/s)	5.04	0.69	3.1	4.5	5	5.4	7.7
β cfPWV	0.67	0.18	0.26	0.54	0.65	0.77	1.45
crPWV (m/s)	7.45	1.48	4.4	6.6	7.5	8.2	12.3
β crPWV	1.48	0.59	0.52	1.12	1.44	1.71	3.74
PWV Ratio	0.68	0.13	0.42	0.61	0.68	0.72	1.05
β PWV Ratio	0.47	0.18	0.17	0.37	0.46	0.53	1.11
**Aortic pressure levels, wave components, reflection and wave-derived parameters**							
aoSBP (RT, SCOR) (mmHg)	83	6	64	78	83	87	100
aoSBP (CT, SCOR) (mmHg)	91	11	69	84	90	97	152
aoSBP (MOG) (mmHg)	86	6	71	82	86	90	101
AIx@75 (RT, SCOR) (%)	17.5	9.3	−10.0	11.0	18.0	24.0	43.0
AIx@75 (CT, SCOR) (%)	−10.4	12.3	−50.0	−18.0	−11.0	−4.0	28.0
AIx@75 (MOG) (%)	28.5	9.6	3.0	22.3	28.1	34.0	65.0
SEVR (RT, SCOR) (%)	116	19	59	101	116	128	179
SEVR (CT, SCOR) (%)	110	18	57	96	110	120	156
aoBPPf (RT, SCOR) (mmHg)	19.9	4.6	7.0	17.0	19.0	23.0	43.0
aoBPPf (CT, SCOR) (mmHg)	31.7	9.7	17.0	25.0	30.0	37.0	76.0
aoBPPf (MOG) (mmHg)	17.6	3.6	10.3	15.0	17.2	19.6	33.6
aoBP Pb (RT, SCOR) (mmHg)	10.0	2.1	3.0	9.0	10.0	11.0	17.0
aoBP Pb (CT, SCOR) (mmHg)	11.2	2.7	7.0	9.0	11.0	13.0	24.0
aoBP Pb (MOG) (mmHg)	9.3	2.1	4.2	7.9	9.2	10.7	17.3

MV: mean value. SD: standard deviation. Min, Max: minimum and maximum. p25th, p50th, p75th: percentiles 25, 50, 75. SBP, DBP, PP: systolic, diastolic and pulse pressure (ao: aortic, ba: brachial, ta: tibial, ra: radial). SysD, DD: peak systolic and end-diastolic diameter. PSV, EDV: peak systolic and end diastolic velocity. IMT: intima–media thickness. BP: blood pressure. EM: elastic modulus. cfPWV, crPWV: carotid-femoral and carotid-radial pulse wave velocity. MOG: Mobil-O-Graph. SCOR: SphygmoCor. PI: Pulsatile Index. Pf, Pb: forward and backward components of aortic blood pressure. RI: resistive index. S-D: Systo-Diastolic index. RT, CT: radial and carotid tonometry. SEVR: subendocardial viability ratio. AIx@75: augmentation index corrected for 75 beats/minute. β: Beta Index.

**Table 3:** Associations between cardiovascular parameters, movement behavior components, CRFs and anthropometric data.

Dependent Variable	Independent Variables	Bu	SE	C.I. LL	C.I. UL	Bs	*p*	VIF	R	R^2^	Adj R^2^
**Central (aortic) and peripheral blood pressure and hemodynamic parameters**											
z-aoDBP (SD)	Constant	−0.172	0.093	−0.355	0.011		0.066		0.416	0.173	0.165
	HBP (1:Yes, 0:No)	1.101	0.154	0.798	1.404	0.362	<0.001	1.007			
	Current z-BW (SD)	0.095	0.039	0.018	0.173	0.122	0.016	1.007			
	**Screen time (min/day)**	**0.001**	**0.000**	**0.000**	**0.002**	**0.121**	**0.017**	**1.006**			
z-baSBP (SD)	Constant	−0.060	0.082	−0.221	0.101		0.463		0.636	0.404	0.399
	HBP (1:Yes, 0:No)	1.938	0.129	1.684	2.192	0.545	<0.001	1.016			
	Current z-BW (SD)	0.164	0.056	0.055	0.273	0.171	0.003	2.562			
	Current z-BH (SD)	0.183	0.068	0.049	0.317	0.154	0.008	2.554			
	**PA, Kinder (min/w)**	**−0.003**	**0.001**	**−0.006**	**−0.001**	**−0.093**	**0.011**	**1.014**			
z-baDBP (SD)	Constant	−0.393	0.091	−0.572	−0.214		<0.001		0.512	0.262	0.258
	HBP (1:Yes, 0:No)	1.621	0.141	1.343	1.899	0.461	<0.001	1.005			
	Current z-BW (SD)	0.153	0.038	0.078	0.228	0.161	<0.001	1.007			
	**Screen time (min/day)**	**0.001**	**0.000**	**0.000**	**0.002**	**0.100**	**0.014**	**1.004**			
z-taSBP (SD)	Constant	0.069	0.118	−0.163	0.301		0.560		0.504	0.254	0.242
	HBP (1:Yes, 0:No)	0.931	0.152	0.632	1.229	0.311	<0.001	1.012			
	Current z-BW (SD)	0.252	0.041	0.171	0.332	0.314	<0.001	1.024			
	**Sedentary play (min/day)**	**−0.002**	**0.001**	**−0.003**	**−0.001**	**−0.146**	**0.004**	**1.016**			
	**PA, Kinder (min/w)**	**−0.004**	**0.002**	**−0.007**	**−0.001**	**−0.148**	**0.004**	**1.029**			
	**PA (min/w)**	**0.000**	**0.000**	**0.000**	**0.001**	**0.118**	**0.021**	**1.023**			
z-taDBP (SD)	Constant	0.080	0.111	−0.139	0.299		0.473		0.470	0.221	0.208
	HBP (1:Yes, 0:No)	1.004	0.169	0.672	1.336	0.311	<0.001	1.029			
	Obesity (1:Yes, 0:No)	0.657	0.148	0.365	0.948	0.231	<0.001	1.029			
	Sex (1:Female, 0:Male)	0.409	0.115	0.182	0.635	0.186	<0.001	1.035			
	**Sedentary play (min/day)**	**−0.002**	**0.001**	**−0.003**	**0.000**	**−0.124**	**0.018**	**1.032**			
	**PA: bike time (min/w)**	**0.002**	**0.001**	**0.000**	**0.005**	**0.109**	**0.037**	**1.021**			
z-taPP (SD)	Constant	−0.021	0.119	−0.255	0.213		0.862		0.310	0.096	0.084
	Sex (1:Female, 0:Male)	−0.393	0.117	−0.625	−0.162	−0.187	0.001	1.020			
	Current z-BW (SD)	0.127	0.046	0.037	0.218	0.154	0.006	1.011			
	**PA: active play (min/w)**	**0.001**	**0.000**	**0.000**	**0.001**	**0.142**	**0.012**	**1.027**			
	**PA, Kinder (min/w)**	**−0.004**	**0.002**	**−0.007**	**0.000**	**−0.117**	**0.036**	**1.014**			
z-raDBP (SD)	Constant	−1.158	0.486	−2.115	−0.201		0.018		0.467	0.218	0.203
	HBP (1:Yes, 0:No)	1.036	0.180	0.680	1.391	0.347	<0.001	1.011			
	Current z-BW (SD)	0.184	0.049	0.087	0.280	0.226	<0.001	1.007			
	Sex (1:Female, 0:Male)	0.249	0.116	0.020	0.477	0.129	0.033	1.001			
	**Sleep (hours/day)**	**0.099**	**0.047**	**0.007**	**0.191**	**0.129**	**0.034**	**1.011**			
z-Ankle-brachial Index (SD)	Constant	0.220	0.112	0.000	0.441		0.051		0.234	0.055	0.045
	**Sedent behavior (min/day)**	**−0.001**	**0.000**	**−0.002**	**0.000**	**−0.161**	**0.005**	**1.001**			
	HBP (1:Yes, 0:No)	−0.302	0.137	−0.573	−0.032	−0.124	0.029	1.001			
	**PA: active play (min/w)**	**0.000**	**0.000**	**0.000**	**0.001**	**0.111**	**0.050**	**1.000**			
z-Cardiac Output (SD)	Constant	0.591	0.342	−0.082	1.264		0.085		0.561	0.315	0.300
	Current z-BW (SD)	0.355	0.071	0.216	0.494	0.538	<0.001	4.501			
	Sex (1:Female, 0:Male)	−0.588	0.088	−0.761	−0.415	−0.340	<0.001	1.012			
	HBP (1:Yes, 0:No)	0.575	0.127	0.326	0.825	0.232	<0.001	1.027			
	Current z-BMI (SD)	−0.335	0.080	−0.494	−0.177	−0.500	<0.001	5.631			
	Obesity (1:Yes, 0:No)	0.637	0.180	0.283	0.991	0.288	<0.001	2.598			
	**Sleep (hours/day)**	**−0.068**	**0.032**	**−0.132**	**−0.005**	**−0.108**	**0.035**	**1.012**			
**Arterial structural parameters**											
z-CCA SystD (SD)	Constant	−0.252	0.097	−0.443	−0.060		0.010		0.479	0.230	0.218
	Current z-BW (SD)	0.265	0.044	0.179	0.351	0.374	<0.001	1.008			
	Sex (1:Female, 0:Male)	−0.370	0.116	−0.598	−0.141	−0.200	0.002	1.052			
	**PA, out kinder (min/w)**	**0.002**	**0.001**	**0.001**	**0.004**	**0.199**	**0.002**	**1.057**			
z-CCA DD (SD)	Constant	−0.252	0.102	−0.453	−0.051		0.014		0.485	0.235	0.224
	Current z-BW (SD)	0.273	0.046	0.183	0.363	0.365	<0.001	1.008			
	Sex (1:Female, 0:Male)	−0.423	0.122	−0.663	−0.183	−0.218	0.001	1.052			
	**PA, out kinder (min/w)**	**0.003**	**0.001**	**0.001**	**0.004**	**0.205**	**0.001**	**1.057**			
z-CCA IMT (SD)	Constant	−0.720	0.158	−1.031	−0.409		<0.001		0.400	0.160	0.148
	Current z-BMI (SD)	0.234	0.061	0.113	0.355	0.245	<0.001	1.009			
	Sex (1:Female, 0:Male)	−0.602	0.161	−0.920	−0.285	−0.240	<0.001	1.003			
	**PA, Kinder (min/w)**	**0.006**	**0.002**	**0.001**	**0.011**	**0.167**	**0.010**	**1.007**			
z-CFA SystD (SD)	Constant	−0.668	0.156	−0.975	−0.361		<0.001		0.521	0.271	0.260
	Current z-BH (SD)	0.446	0.074	0.300	0.591	0.369	<0.001	1.004			
	Sex (1:Female, 0:Male)	−0.640	0.152	−0.939	−0.341	−0.261	<0.001	1.034			
	**PA: typical week (min/w)**	**0.001**	**0.000**	**0.000**	**0.001**	**0.202**	**0.001**	**1.037**			
z-CFA DD (SD)	Constant	−0.547	0.159	−0.861	−0.232		0.001		0.502	0.252	0.241
	Current z-BH (SD)	0.450	0.075	0.301	0.598	0.368	<0.001	1.004			
	Sex (1:Female, 0:Male)	−0.607	0.155	−0.912	−0.301	−0.245	<0.001	1.034			
	**PA: typical week (min/w)**	**0.001**	**0.000**	**0.000**	**0.001**	**0.183**	**0.004**	**1.037**			
z-BA SystD (SD)	Constant	−0.759	0.098	−0.954	−0.565		<0.001		0.485	0.235	0.217
	Current z-BW (SD)	0.406	0.078	0.252	0.560	0.627	<0.001	2.459			
	**PA: walk time (min/w)**	**0.002**	**0.001**	**0.001**	**0.003**	**0.246**	**0.002**	**1.008**			
	Obesity (1:Yes, 0:No)	−0.690	0.256	−1.196	−0.185	−0.325	0.008	2.465			
z-BA DD (SD)	Constant	−0.715	0.104	−0.920	−0.510		<0.001		0.477	0.227	0.209
	Current z-BW (SD)	0.419	0.082	0.256	0.581	0.617	<0.001	2.459			
	**PA: walk time (min/w)**	**0.002**	**0.001**	**0.001**	**0.003**	**0.235**	**0.003**	**1.008**			
	Obesity (1:Yes, 0:No)	−0.692	0.269	−1.225	−0.160	−0.311	0.011	2.465			
**Blood flow velocity and Doppler-derived parameters**											
z-CCA EDV (SD)	Constant	0.648	0.108	0.434	0.861		<0.001		0.252	0.063	0.054
	Current z-BW (SD)	−0.153	0.053	−0.258	−0.048	−0.197	0.004	1.005			
	**PA, Kinder (min/w)**	**−0.004**	**0.002**	**−0.008**	**0.000**	**−0.145**	**0.035**	**1.005**			
z-CCA RI (SD)	Constant	−0.531	0.100	−0.728	−0.334		<0.001		0.299	0.090	0.080
	Current z-BH (SD)	0.187	0.057	0.074	0.300	0.220	0.001	1.004			
	**PA: typical week (min/w)**	**0.001**	**0.000**	**0.000**	**0.001**	**0.189**	**0.005**	**1.004**			
z-CCA PI (SD)	Constant	−0.784	0.088	−0.957	−0.611		<0.001		0.380	0.144	0.132
	Current z-BW (SD)	0.155	0.039	0.078	0.232	0.261	<0.001	1.012			
	**PA: typical week(min/w)**	**0.001**	**0.000**	**0.000**	**0.001**	**0.270**	**<0.001**	**1.060**			
	**PA: bike time (min/w)**	**−0.002**	**0.001**	**−0.004**	**0.000**	**−0.134**	**0.048**	**1.066**			
z-CCA S-D Index (SD)	Constant	−0.549	0.091	−0.729	−0.370		<0.001		0.346	0.120	0.111
	Current z-BH (SD)	0.195	0.052	0.093	0.298	0.248	<0.001	1.004			
	**PA: typical week (min/w)**	**0.001**	**0.000**	**0.000**	**0.001**	**0.226**	**0.001**	**1.004**			
z-CFA PSV (SD)	Constant	−0.304	0.125	−0.550	−0.059		0.015		0.304	0.092	0.078
	**PA: walk time (min/w)**	**0.002**	**0.001**	**0.000**	**0.003**	**0.164**	**0.019**	**1.016**			
	Sex (1:Female, 0:Male)	0.365	0.143	0.082	0.648	0.176	0.012	1.003			
	HBP (1:Yes, 0:No)	0.445	0.197	0.056	0.833	0.157	0.025	1.013			
z-CFA RI (SD)	Constant	0.046	0.140	−0.229	0.322		0.741		0.380	0.145	0.131
	Obesity (1:Yes, 0:No)	−0.861	0.207	−1.271	−0.452	−0.283	<0.001	1.033			
	HBP (1:Yes, 0:No)	−0.567	0.227	−1.014	−0.120	−0.170	0.013	1.031			
	**Sedent play (min/day)**	**0.002**	**0.001**	**0.000**	**0.004**	**0.148**	**0.029**	**1.006**			
z-CFA S-D Index (SD)	Constant	−3.661	1.571	−6.760	−0.563		0.021		0.239	0.057	0.047
	**PA: bike time (min/w)**	**0.005**	**0.002**	**0.002**	**0.009**	**0.203**	**0.005**	**1.016**			
	Age (years)	0.595	0.273	0.056	1.134	0.154	0.031	1.016			
z-BA EDV (SD)	Constant	−0.081	0.098	−0.275	0.112		0.409		0.384	0.148	0.122
	**Screen time (min/day)**	**−0.001**	**0.000**	**−0.002**	**0.000**	**−0.234**	**0.005**	**1.026**			
	**PA, out kinder (min/w)**	**−0.002**	**0.001**	**−0.003**	**0.000**	**−0.206**	**0.012**	**1.012**			
	Current z-BMI (SD)	0.248	0.080	0.089	0.406	0.572	0.002	5.340			
	Current z-BW (SD)	−0.185	0.080	−0.344	−0.026	−0.426	0.023	5.341			
**Aortic pressure levels, wave components, reflection and wave-derived parameters**											
z-aoSBP (RT) (SD)	Constant	−2.169	0.865	−3.874	−0.464		0.013		0.576	0.332	0.320
	HBP (1:Yes, 0:No)	1.206	0.153	0.905	1.507	0.436	<0.001	1.004			
	Current z-BW (SD)	0.232	0.041	0.152	0.312	0.315	<0.001	1.009			
	**PA, Kinder (min/w)**	**−0.004**	**0.001**	**−0.007**	**−0.001**	**−0.154**	**0.008**	**1.068**			
	Age (years)	0.395	0.153	0.094	0.695	0.147	0.010	1.062			
z-AIx@75 (CT) (SD)	Constant	−0.509	0.149	−0.804	−0.214		0.001		0.440	0.194	0.176
	Sex (1:Female, 0:Male)	0.744	0.157	0.434	1.055	0.321	<0.001	1.021			
	Current z-BW (SD)	−0.240	0.067	−0.372	−0.107	−0.240	<0.001	1.002			
	**Sedent play (min/day)**	**−0.002**	**0.001**	**−0.004**	**0.000**	**−0.171**	**0.013**	**1.023**			
	z-BWH birth (SD)	0.165	0.073	0.022	0.309	0.153	0.024	1.004			
z-AIx@75 (MOG) (SD)	Constant	0.209	0.134	−0.054	0.472		0.119		0.477	0.227	0.216
	Sex (1:Female, 0:Male)	0.810	0.127	0.560	1.060	0.343	<0.001	1.002			
	Current z-BW (SD)	−0.248	0.049	−0.344	−0.152	−0.275	<0.001	1.007			
	HBP (1:Yes, 0:No)	0.459	0.182	0.101	0.817	0.136	0.012	1.008			
	**Screen time (min/day)**	**0.001**	**0.001**	**0.000**	**0.003**	**0.136**	**0.012**	**1.009**			
z-SEVR (CT) (SD)	Constant	−0.931	0.152	−1.231	−0.631		<0.001		0.400	0.160	0.142
	**PA: typical week (min/w)**	**0.001**	**0.000**	**0.000**	**0.001**	**0.218**	**0.002**	**1.022**			
	HBP (1:Yes, 0:No)	−0.621	0.225	−1.064	−0.178	−0.185	0.006	1.000			
	z-BWH birth (SD)	0.189	0.071	0.050	0.328	0.180	0.008	1.005			
	Sex (1:Female, 0:Male)	−0.381	0.151	−0.678	−0.084	−0.171	0.012	1.017			
z-aoBPPf (CT) (SD)	Constant	−4.335	2.261	−8.808	0.138		0.057		0.460	0.212	0.182
	Age (years)	1.156	0.368	0.428	1.884	0.246	0.002	1.014			
	HBP (1:Yes, 0:No)	1.282	0.395	0.501	2.063	0.257	0.001	1.030			
	**PA: bike time (min/w)**	**0.005**	**0.002**	**0.001**	**0.009**	**0.210**	**0.009**	**1.034**			
	**Sleep (hours/day)**	**−0.187**	**0.087**	**−0.360**	**−0.014**	**−0.168**	**0.034**	**1.019**			
	Current z-BH (SD)	0.224	0.113	0.001	0.447	0.157	0.049	1.023			

z: z-score. SBP, DBP, PP: systolic, diastolic and pulse pressure (ao: aortic, ba: brachial, ta: tibial, ra: radial). SysD, DD: peak systolic and end-diastolic diameter, respectively. RI, PI: resistive and pulsatile index. S-D: Systo-Diastolic. IMT: intima–media thickness. PSV, EDV: blood flow peak systolic and end-diastolic velocity. MOG: Mobil-O-Graph. RT, CT: radial and carotid tonometry. SEVR: subendocardial viability ratio. AIx@75: augmentation index for 75 beats/minute. Pf: forward pressure wave components. HBP: high blood pressure. PA: physical activity. BW, BH: body weight and height. BMI: body mass index. Bu y Bs: un- and standardized coefficients. R: Pearson coefficient. Adj: adjusted. VIF: variance inflation factor. SE: standard error. L.L, U.L: Lower and upper limit. C.I: 95% confidence interval. w: week. Only significant (*p* < 0.05) variables entered in the models are shown. Bold: movement behavior component or subcomponent.

**Table 4:** Impact of interindividual variations in MB, on central and peripheral blood pressure, cardiac output and arterial characteristics.

Dependent Variable			Cardiovascular Variations Related to Movement Behavior Parameters Variations									
Variable	MV (RG)	SD (RG)	Modeled Variation (Δ Minutes):	60	120	180	240	300	Min	Max	Δ	Δ%
**Central (aortic) and peripheral blood pressure and cardiac output**												
aoDBP (mmHg)	59.2	5.4	Screen time (m/d)	0.4	0.7	1.1	1.5	1.8	0	3.6	3.6	6.1
baSBP (mmHg)	98.4	5.2	PA, Kinder (m/w)	−1.1	−2.1	−3.2	−4.2	−5.3	0	−1.59	1.59	1.62
baDBP (mmHg)	58.8	3.9	Screen time (m/d)	0.3	0.5	0.8	1.1	1.4	0	2.71	2.71	4.60
taSBP (mmHg)	113.6	7.5	Sedent. play (m/d)	−0.8	−1.6	−2.3	−3.1	−3.9	0	−6.25	6.25	5.50
			PA, Kinder (m/w)	−2.0	−4.0	−6.0	−8.0	−10.0	0	−3.01	3.01	2.65
			PA, typical week (m/w)	0.2	0.3	0.5	0.6	0.8	0	4.58	4.58	4.03
taDBP (mmHg)	58.1	5.3	Sedent. play (m/d)	−0.5	−1.0	−1.5	−2.0	−2.5	0	−4.05	4.05	6.97
			PA: bike time (m/w)	0.8	1.6	2.4	3.1	3.9	0	3.27	3.27	5.63
taPP (mmHg)	55.5	7.0	PA: active play (m/w)	0.2	0.5	0.7	0.9	1.1	0	4.55	4.55	8.20
			PA, Kinder (m/w)	−1.5	−3.1	−4.6	−6.1	−7.7	0	−2.30	2.30	4.14
raDBP (mmHg)	57.6	5.7	Sleep (h/d)	0.6	1.1	1.7	2.3	2.8	3.95	9.58	5.64	9.79
Ankle Brachial Index	1.15	0.09	Sedent. behavior (m/d)	−0.01	−0.01	−0.02	−0.02	−0.03	0	−0.06	0.06	5.43
			PA: active play (m/w)	0.00	0.00	0.01	0.01	0.01	0	0.04	0.04	3.21
CO (L/min)	4.41	0.54	Sleep (h/d)	−0.04	−0.07	−0.11	−0.15	−0.19	−0.26	−0.63	0.37	8.41
**Arterial structural parameters**												
CCA SystD (mm)	6.05	0.49	PA, out Kinder (m/w)	0.07	0.15	0.22	0.29	0.37	0	0.32	0.32	5.25
CCA DD (mm)	5.38	0.43	PA, out Kinder (m/w)	0.07	0.14	0.21	0.28	0.35	0	0.30	0.30	5.66
CCA IMT (mm)	0.43	0.02	PA, Kinder (m/w)	0.01	0.01	0.02	0.03	0.04	0	0.01	0.01	2.52
CFA SystD (mm)	5.01	0.40	PA: typical week (m/w)	0.02	0.04	0.05	0.07	0.09	0	0.54	0.54	10.81
CFA DD (mm)	4.64	0.40	PA: typical week (m/w)	0.02	0.03	0.05	0.06	0.08	0	0.49	0.49	10.49
BA SystD (mm)	2.66	0.37	PA: walk time (m/w)	0.04	0.08	0.12	0.15	0.19	0	0.32	0.32	12.09
BA DD (mm)	2.45	0.35	PA: walk time (m/w)	0.04	0.07	0.11	0.15	0.19	0	0.31	0.31	12.59
**Blood flow velocity and Doppler-derived parameters**												
CCA EDV (cm/s)	31.68	6.28	PA, Kinder (m/w)	−1.63	−3.26	−4.90	−6.53	−8.16	0	−2.45	2.45	7.73
CCA RI	0.75	0.05	PA: typical week (m/w)	0.00	0.00	0.00	0.01	0.01	0	0.04	0.04	5.85
CCA PI	1.86	0.35	PA: typical week (m/w)	0.01	0.03	0.04	0.05	0.07	0	0.40	0.40	21.44
			PA: bike time (m/w)	−0.05	−0.09	−0.14	−0.19	−0.23	0	−0.19	0.19	10.40
CCA S-D Index	4.11	0.81	PA: typical week (m/w)	0.03	0.05	0.08	0.11	0.14	0	0.82	0.82	19.97
CFA PSV (cm/s)	122.64	23.32	PA: walk time (m/w)	2.11	4.22	6.32	8.43	10.54	0	17.57	17.57	14.32
CFA RI	0.97	0.08	Sedent. play (m/d)	0.01	0.02	0.03	0.04	0.05	0	0.07	0.07	7.56
CFA S-D Index	34.04	33.03	PA: bike time (m/w)	10.59	21.17	31.76	42.35	52.93	0	44.11	44.11	129.57
BA EDV (cm/s)	13.21	14.07	Screen time (m/d)	−1.11	−2.21	−3.32	−4.42	−5.53	0	−11.05	11.05	83.64
			PA, out Kinder (m/w)	−1.44	−2.88	−4.32	−5.76	−7.20	0	−6.24	6.24	47.22
**Aortic pressure levels, wave components, reflection and wave-derived parameters**												
aoSBP (RT) (mmHg)	81.00	7.06	PA, Kinder (m/w)	−1.68	−3.36	−5.04	−6.72	−8.39	0	−2.52	2.52	3.11
AIx@75 (CT) (%)	−5.18	11.09	Sedent. play (m/d)	−1.48	−2.97	−4.45	−5.93	−7.41	0	−11.86	11.86	228.94
AIx@75 (MOG) (%)	21.81	8.28	Screen time (m/d)	0.73	1.45	2.18	2.91	3.63	0	7.27	7.27	33.32
SEVR (CT) (%)	122.18	15.70	PA: typical week (m/w)	0.67	1.33	2.00	2.66	3.33	0	19.98	19.98	16.36
aoBPPf (CT) (mmHg)	28.22	6.42	PA: bike time (m/w)	2.07	4.14	6.22	8.29	10.36	0	8.63	8.63	30.59
			Sleep (h/d)	−1.20	−2.40	−3.60	−4.80	−6.00	−8.40	−20.40	12.00	42.52

z: z-score. RG: reference group. MV: mean value. SD: standard deviation. Min, Max: minimum and maximum. SBP, DBP, PP: systolic, diastolic and pulse pressure (ao: aortic, ba: brachial, ta: tibial, ra: radial). SysD, DD: peak systolic and end-diastolic diameter. RI, PI: resistive and pulsatile index. S-D: Systo-Diastolic. IMT: intima–media thickness. PSV, EDV: peak systolic and end diastolic velocity. MOG: Mobil-O-Graph. CO: cardiac output. RT, CT: radial and carotid tonometry. SEVR: subendocardial viability ratio. AIx@75: augmentation index for 75 beats/minute. Pf: forward aortic pressure wave components. PA: physical activity. (m/d): minute/day. (m/w): minute/week. (h/d): hours/day. Δ was calculated as: (Max−Min) [net value]. Δ% was calculated as: ((Max−Min)/MV) × 100. Kinder: at kindergarten. PA out of Kinder: Physical activity outside of kindergarten. Sedent.: Sedentary.

## Data Availability

The data presented in the study are available within the article and in Supplementary Material.

## Supplementary Materials

The following are available online, Table S1, Hemodynamic, structural and functional arterial parameters of preschool-aged children: Reference Subgroup (subjects non-exposed to CRFs and who complied with 24 h movement recommendations). Table S2, Hemodynamic, structural and functional cardiovascular parameters z-scores of preschool-aged children (Reference subgroup). Table S3, Hemodynamic, structural and functional cardiovascular parameters z-scores of preschool-aged children (Allchildren). Table S4, Association between cardiovascular z-scores (dependent variables) and triad, CRF and anthropometric parameters (independent variables) in preschool-aged children. Table S5, Association between physical activity, sedentary behavior and sleep time components and sub-components. Table S6, Association between movement behavior and factors related to increased cardiovascular risk. Table S7, Central and peripheral blood pressure and vascular parameters: association with factors related to increased cardiovascular risk. Table S8, Association between blood pressure and structural and functional arterial parameters z-scores with movement behaviors components and sub-components. Table S9, Association between blood pressure and structural and functional arterial parameters z-scores with cardiovascular risk factors. Table S10, Impact of interindividual variations in movement behaviors (independent variables) on cardiovascular characteristics (dependent variables), in preschool-aged children.

## Abbreviations

ABI	Ankle Brachial index
AIx@75	AIx adjusted to a 75 beats/min heart rate
aoBP	Central (aortic) blood pressure
aoDBP	Aortic diastolic blood pressure
aoPP	Aortic pulse pressure
aoSBP	Aortic systolic blood pressure
BA	Brachial artery
baBP	Brachial artery blood pressure
baDBP	Brachial artery (peripheral) diastolic blood pressure
baPP	Brachial artery pulse pressure
baSBP	Brachial artery (peripheral) systolic blood pressure
BH	Body height
BMI	Body mass index
BP	Blood pressure
BW	Body weight
CCA	Common carotid artery
CFA	Common femoral artery
cfPWV	Carotid-femoral pulse wave velocity
CRFs	Cardiovascular risk factors
crPWV	Carotid-radial pulse wave velocity
CV	Cardiovascular
DD	End-diastolic arterial diameter
EDV	End-diastolic blood flow velocity
EM	Pressure-strain or Petrson arterial elastic modulus
IMT	Intima-media thickness
MB	Movement behavior
MBP	Brachial artery mean blood pressure
MET	Metabolic equivalent
MOG	Mobil-O-Graph PWA-monitor device (MOG; I.E.M.-GmbH, Stolberg, Germany)
PA	Physical activity
Pb	Amplitude of the aortic pressure waveform backward component
Pf	Amplitude of the aortic pressure waveform forward component
PSV	Peak systolic blood flow velocity
raDBP	Radial artery diastolic blood pressure
raPP	Radial artery pulse pressure
raSBP	Radial artery systolic blood pressure
SB	Sedentary behavior
SCOR	SphygmoCor-CvMS device (SCOR; v.9, AtCor-Medical, Australia)
ST	Sleep time
SystD	Peak systolic arterial diameter
taDBP	Tibial artery diastolic blood pressure
taPP	Tibial artery pulse pressure
taSBP	Tibial artery systolic blood pressure
β	Beta or stiffness (local) index
